# Two-photon excited fluorescence of intrinsic fluorophores enables label-free assessment of adipose tissue function

**DOI:** 10.1038/srep31012

**Published:** 2016-08-05

**Authors:** Carlo Amadeo Alonzo, Sevasti Karaliota, Dimitra Pouli, Zhiyi Liu, Katia P. Karalis, Irene Georgakoudi

**Affiliations:** 1 Department of Biomedical Engineering, Tufts University 4 Colby Street, Medford, MA 02155, USA; 2 Biomedical Research Foundation, Academy of Athens, Athens, Greece; 3 Endocrine Division, Children’s Hospital, Boston, MA 02115, USA.

## Abstract

Current methods for evaluating adipose tissue function are destructive or have low spatial resolution. These limit our ability to assess dynamic changes and heterogeneous responses that occur in healthy or diseased subjects, or during treatment. Here, we demonstrate that intrinsic two-photon excited fluorescence enables functional imaging of adipocyte metabolism with subcellular resolution. Steady-state and time-resolved fluorescence from intracellular metabolic co-factors and lipid droplets can distinguish the functional states of excised white, brown, and cold-induced beige fat. Similar optical changes are identified when white and brown fat are assessed *in vivo*. Therefore, these studies establish the potential of non-invasive, high resolution, endogenous contrast, two-photon imaging to identify distinct adipose tissue types, monitor their functional state, and characterize heterogeneity of induced responses.

Adipose tissue function has been recognized to exert significant influence on systemic metabolic balance and overall homeostasis health through energy storage via lipid accumulation, direct energy expenditure through substrate oxidation^1^,^2^, and secretion of various signaling and regulatory molecules^3^,^4^. Major health problems such as type 2 diabetes mellitus, cancer, and cardiovascular disease are linked to dysregulation of endocrine function due to excessive lipid accumulation in adipose tissue, i.e. obesity^5^. Intrinsic cellular metabolism is a key indicator of healthy function in distinct adipose tissue types across their different roles. For example, white adipose tissue (WAT) favors lipogenesis pathways towards accumulation of triacylglycerol (TAG) in large lipid droplets (LD) within the adipocytes for energy storage, but also maintains a dynamic balance with lipolysis of TAG for both signaling and energy mobilization functions^3^. Adipocytes in brown adipose tissue (BAT) feature smaller intracellular LDs and favor metabolic pathways involving oxidation of glucose and fatty acids to fuel heat energy dissipation in mitochondria^2^,^6^. Notably, this thermogenic function is most conspicuous in small mammals, including human infants. Only recently have studies found that adult humans maintain some capacity for thermogenesis in adipose tissue^7^,^8^. Potentiation of this response through environmental and pharmacological stimuli has garnered wide interest as a therapeutic target for obesity and its metabolic health complications^9^. However, assessments of the efficacy of such interventions are limited in their ability to characterize the both dynamic and heterogeneous functional responses in adipose tissue. For example, certain stimuli can induce “browning” of WAT in some fat depots into beige adipose tissue (BeAT) forming isolated clusters of thermogenic BAT-like adipocytes^10^,^11^. The standard analysis tool for adipose tissue function, gene expression profiling of molecular markers, is very sensitive, but is also destructive. Thus, it is not well suited to study dynamic changes. Positron emission tomography (PET) provides real-time measurement of metabolic activity, but its millimeter-scale spatial resolution still cannot account for cellular heterogeneity^7^,^8^. Consequently, there is a need for non-destructive approaches that can dynamically assess adipose tissue function at the cellular level.

Two-photon excited fluorescence (TPEF) microscopy of intracellular coenzymes, flavin adenine dinucleotide (FAD) and pyridine nucleotides (NAD(P)H), is a high-resolution imaging technique that provides non-destructive dynamic assessment of cellular metabolism^12^,^13^,^14^. Both steady-state and time-resolved fluorescence intensity of these coenzymes can be probed to yield complementary information^15^. The steady-state fluorescence intensity ratio between FAD and NAD(P)H correlates to the oxidation-reduction state of cells, and is commonly referred to as an optical redox ratio^16^,^17^. This redox ratio has been shown to be a sensitive biomarker of changes associated with the relative rates of key metabolic and biosynthetic pathways, including glycolysis, oxidative phosphorylation, glutaminolysis, and lipid synthesis^18^,^19^. Shifts in cellular metabolic pathway utilization also lead to alterations in the rate and distribution of NAD(P)H binding to different oxidoreductase enzymes, leading to distinct NAD(P)H fluorescence lifetimes^20^,^21^,^22^,^23^. Fluorescence lifetime imaging has been used to characterize metabolic changes in cancer cells^14^,^15^ and differentiating stem cells^18^,^23^,^24^,^25^. Recently, intrinsic TPEF from LDs has also been reported^26^,^27^, but LD fluorescence differences between different adipose tissue types has not yet been explored.

Our goal for this study is to demonstrate that TPEF microscopy of intrinsic fluorophores enables dynamic and non-destructive assessment of adipose tissue function through functional metabolic imaging with subcellular resolution. In particular, we capture high-resolution three-dimensional images to show that optical redox ratio and fluorescence lifetimes in the cytoplasm can reveal metabolic differences associated with thermogenic function of adipose tissue excised from mice. Such differences are sensitive to thermogenic activation not only of BAT, but also of BeAT. More heterogeneous response within BeAT illustrates the need for high-resolution imaging approaches to more fully understand dynamic tissue function. We also identify differences in the endogenous fluorescence lifetime of LDs that distinguish adipose tissue types and responses to cold activation. This represents an additional potential biomarker of adipose tissue function. We further show that these techniques are translatable *in vivo* by capturing functional TPEF images from BAT and WAT in live mice.

## Results

### TPEF enables high-resolution three-dimensional (3D) imaging of subcellular compartments using only intrinsic contrast

**Figure 1:**
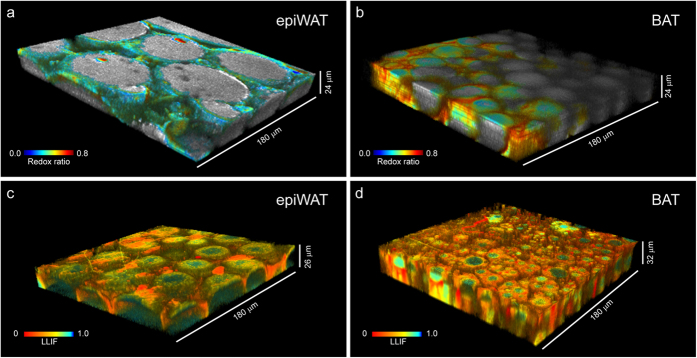
Two-photon excited fluorescence microscopy enables 3D imaging of subcellular compartments in adipose tissue using only intrinsic contrast. (**a,b**) Redox ratio is a ratio of fluorescence intensity from endogenous metabolic co-factors FAD and NADH in the cytoplasm. Lipid droplets, coded in gray, are identified by spectral emission similar to NADH, but not FAD. (**c,d**) Lipid compartments are also contrasted from cytoplasm by longer fluorescence lifetimes (ex755/em460), presented here as the fractional intensity contribution of a 6.5 ns lifetime component. Both modes clearly depict larger lipid droplet size in epididymal white adipose tissue (eWAT) compared to brown adipose tissue (BAT).

Using distinct excitation and emission bandwidths, we isolated TPEF signals from the endogenous metabolic co-factors NAD(P)H (excitation: 755 nm, emission: 460 ± 20 nm) and FAD (excitation: 860 nm, emission: 525 ± 25 nm)^18^. Images were acquired from as deep as 50 μm in tissue excised from the interscapular BAT and epididymal (epiWAT) fat depots of mice. Tissues were frozen for transport and storage, but not otherwise processed. We constructed 3D maps of the optical redox ratio using a ratio of fluorescence intensities: FAD/(NAD(P)H+FAD) (Fig. 1a,b). This form of the redox ratio is normally distributed between the values of 0 and 1, and is strongly correlated with liquid chromatography-mass spectrometry based concentration assessments of FAD/(NADH+FAD), as well as NAD^+^/(NADH+NAD^+^), for both epithelial cells and adipogenically differentiating stem cells^18^,^19^. Cytoplasm was identified by detectable signal in both fluorescence channels, while LDs were distinguished by weak to absent emission at the FAD channel. Redox ratios were not calculated within lipid segments. Instead, LDs are shown in grayscale representing relative intensity at 460 nm. Time-resolved fluorescence at the NAD(P)H/LD fluorescence channel yielded further contrast between cellular compartments. LDs presented longer fluorescence lifetimes (~6.5 ns) compared to the cytoplasm (ranging from 1 to 3 ns) (Fig. 1c,d). We employed the phasor approach to fluorescence lifetime analysis^23^ and coded the images by the fractional contribution to emission intensity from the long lifetime component of a biexponential decay^28^. On this scale, fluorescence lifetime increases as the long lifetime intensity fraction (LLIF) approaches 1 (Supplementary Fig. 1). Volume reconstructions in Fig. 1 emphasize that these two fully intrinsic contrast scales provide high-resolution 3D visualization of cellular morphology that highlight subcellular compartments within adipose tissue. This allows, for example, the identification of larger LD sizes in WAT compared to BAT.

### Functional imaging of cellular metabolism distinguishes thermogenic from non-thermogenic adipose tissue

**Figure 2:**
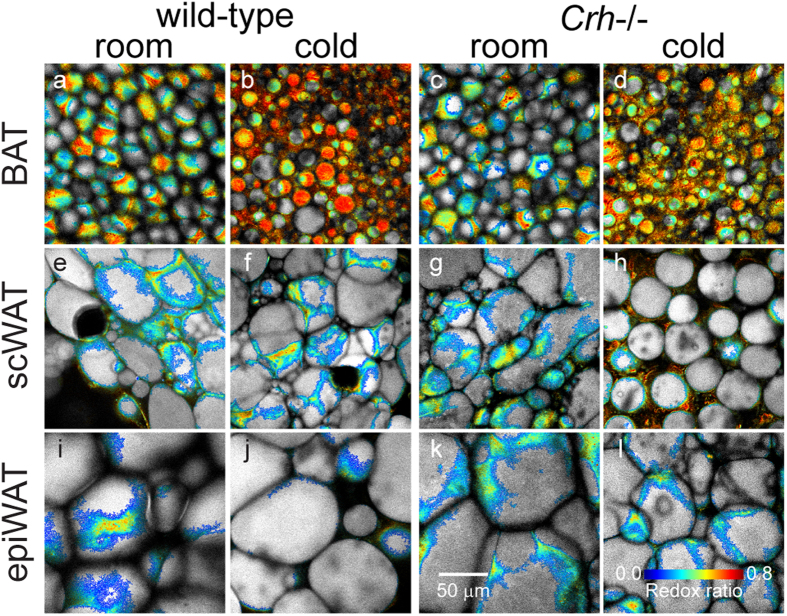
Redox ratio provides functional contrast of cellular metabolism between adipose tissue types. Representative images show mitochondria-rich BAT exhibit higher redox than WAT in subcutaneous inguinal (scWAT) and epididymal (epiWAT) depots at room temperature. Cold activation of thermogenic uncoupling in BAT leads to further increases in redox ratio, but no change in epiWAT. Redox also reveals cold activation in scWAT indicative of tissue browning in *Crh*−/− mice, but not in wild-type mice.

**Figure 3:**
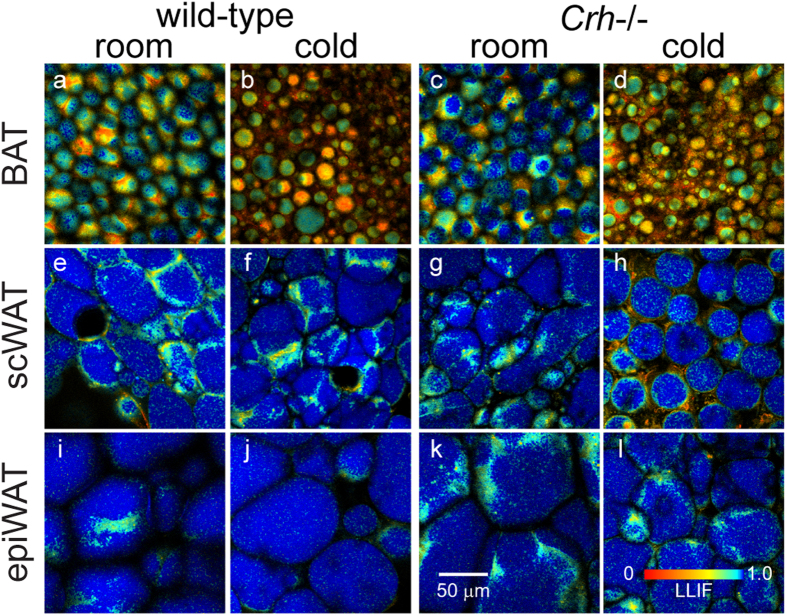
Fluorescence lifetime (ex755/em460) provides additional contrast in adipose tissue. Application of a complimentary color scale (red for short lifetime, blue for long) contrasts similar features as redox ratio images. Lifetime trends parallel those of redox: shorter lifetimes in BAT than scWAT and epiWAT. Cold-activated thermogenesis is also associated with decreases in lifetime in *Crh*−/− scWAT and BAT in both genotypes.

We acquired redox and fluorescence lifetime images from the interscapular BAT, subcutaneous inguinal WAT (scWAT), and epididymal WAT (epiWAT) from each of n = 3 mice (C57BL/6 lineage). Individual image fields were captured from between 6 to 12 separate locations across each ~2 mm × 5 mm tissue specimen to better sample any larger scale tissue heterogeneity. Image stacks were not acquired in this case due to imaging time constraints. In addition, we compared the optical metabolic readouts of these depots from mice housed at room temperature to corresponding depots from mice exposed to 4 °C for 2 days. Interscapular fat in mice is recognized as classical BAT with robust thermogenic response to cold. EpiWAT is often taken as a non-thermogenic control tissue as it is resistant to tissue browning^10^,^11^. In contrast, scWAT exhibits BeAT formation in some mouse strains, although such susceptibility is low in C57BL/6 mice^11^. Lipidomics analysis via liquid chromatography and tandem mass spectrometry (LC-MS/MS) of BAT from the same depots examined under TPEF imaging was consistent with enhanced lipolysis and mitogenesis – markers of thermogenic activation^1^ – in response to cold exposure, while no cold-response was observed in the WAT samples (Supplementary Fig. 2). Smaller-sized LDs in BAT (Fig. 2a) compared to scWAT (Fig. 2e) and epiWAT (Fig. 2i) were immediately evident. More importantly, the images showed higher redox ratio, i.e. more oxidized state, in BAT (yellow-orange) compared to both WAT depots (cyan). This is a reflection of both higher mitochondrial density, and higher turnover rates in the electron transport chain (ETC) in BAT compared to WAT. Consistent with greater engagement of the ETC during thermogenesis, even higher redox was observed in BAT from cold exposed mice (Fig. 2b) compared to BAT from mice kept at room temperature (Fig. 2a). Meanwhile, there were no obvious temperature effects in either WAT depot (Fig. 2f,j). Contrast between adipose tissue depots was further demonstrated in a transgenic mouse model with increased cold-induced BeAT formation in scWAT, the corticotropin-releasing hormone gene knockout (*Crh*−/−) mice (Supplementary Information, Supplementary Fig. 3). This mutation impacts the systemic metabolism of the animals, which exhibit extensive beige adipose tissue in their scWAT contributing both to thermogenesis and to the total body energy expenditure^29^. Thus, it is not surprising that the base redox states of adipose tissue are different between the genotypes. Again, increased redox ratio with cold exposure was evident in the BAT (Fig. 2c,d), but also in some scWAT images (Fig. 2g,h). Redox in epiWAT remained insensitive to temperature (Fig. 2k,l). Parallel to increases in redox, BAT showed lower NAD(P)H fluorescence lifetime (Fig. 3a–d), corresponding to greater ETC activity, relative to scWAT (Fig. 3e–h) and epiWAT (Fig. 3i–l). Further lifetime decreases were also observable with cold exposure in BAT (Fig. 3b,d) and some *Crh*−/− scWAT (Fig. 3h). Taken together, these images demonstrate that functional metabolic imaging via TPEF of intrinsic FAD and NAD(P)H not only provides contrast between different functional types of adipose tissue, i.e. BAT vs. WAT, but can also indicate activation of thermogenic function in BAT and BeAT.

### BAT and WAT present distinctive fluorescence lifetime signatures

**Figure 4:**
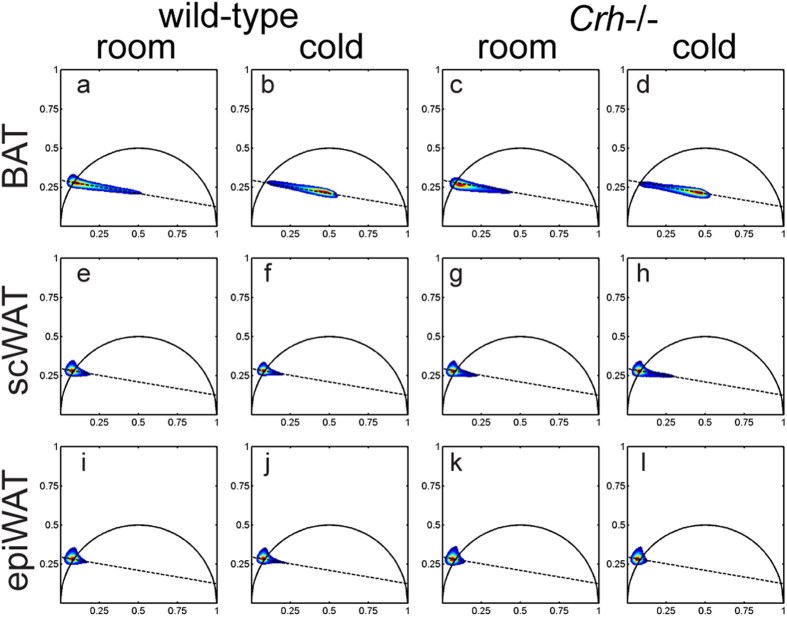
Pixel-by-pixel fluorescence lifetime reveal distinct signatures of WAT and BAT. (**a–d**) Fluorescence lifetime phasor distribution profiles of BAT are spread across a linear trajectory corresponding to a biexponential decay with lifetime components 6.5 ns and 0.3 ns. (**e**–**l**) scWAT and epiWAT distributions are more sharply clustered at the long-lifetime end of the same trajectory. Each panel depicts the peak normalized phasor distribution averaged from 3 mice, based on 6 to 15 acquired images per mouse.

We applied Fourier-based phasor transforms on time-resolved fluorescence data to calculate phasor coordinates describing fluorescence decay at each image pixel^23^. In phasor space, pure monoexponential decays map onto a reference semi-circle, while multiexponentials fill the area within the semi-circle. Fluorescence lifetime increases in the counter-clockwise direction along the reference arc (Supplementary Fig. 1). Cumulative phasor distributions were compiled for each adipose tissue type. WAT depots (Fig. 4e–l) featured sharp distributions clustered about a point corresponding to a lifetime of 6.5 ns on the reference arc. In contrast, BAT phasor distributions (Fig. 4a–d) stretched broadly along a linear trajectory toward a second reference lifetime of 0.3 ns. A significant change in the centroid of the BAT lifetime phasor distribution was detected upon cold activation of both mice strains. Linear trajectories in phasor space suggest biexponential decays featuring the two reference end-points as components. Relative distances to each reference point directly represent fractional contributions of each component to total emission intensity^28^. We used the long lifetime intensity fraction (LLIF) scale to code fluorescence lifetime images (Figs 1 and 3) and found LDs, particularly in WAT, were primarily associated with the 6.5 ns monoexponential decay. Previous reports observed a similar monoexponential profile (7.8 ns) for LD fluorescence in visceral fat of mice^27^. Meanwhile, the short lifetime component of our phasor trajectory falls within the range of lifetimes associated with NAD(P)H free in solution, i.e. 0.2 ns to 0.4 ns^14^,^21^,^22^,^27^. Bound NAD(P)H is associated with a very broad range of longer lifetime components, from 0.8 ns to 5.7 ns depending on the binding enzyme^20^,^21^,^22^,^27^, and even reaching 6.5 ns to 9.5 ns in the presence of substrates^30^,^31^. In general, we would expect NAD(P)H and LD fluorescence phasors to follow independent trajectories. The observed linear, rather than bifurcated, phasor trajectory suggests a high degree of overlap between LD and bound NAD(P)H signatures that our system cannot resolve. Notably, phasor distributions of digitally segmented cytoplasm and LD compartments still follow the same linear trajectories (Supplementary Figs 4 and 5). Despite such overlap, phasor analysis of fluorescence lifetime provides distinguishing signatures between BAT and WAT.

### Endogenous fluorescence of both cytoplasm and lipid droplet compartments reveal differences in adipose tissue function

**Figure 5:**
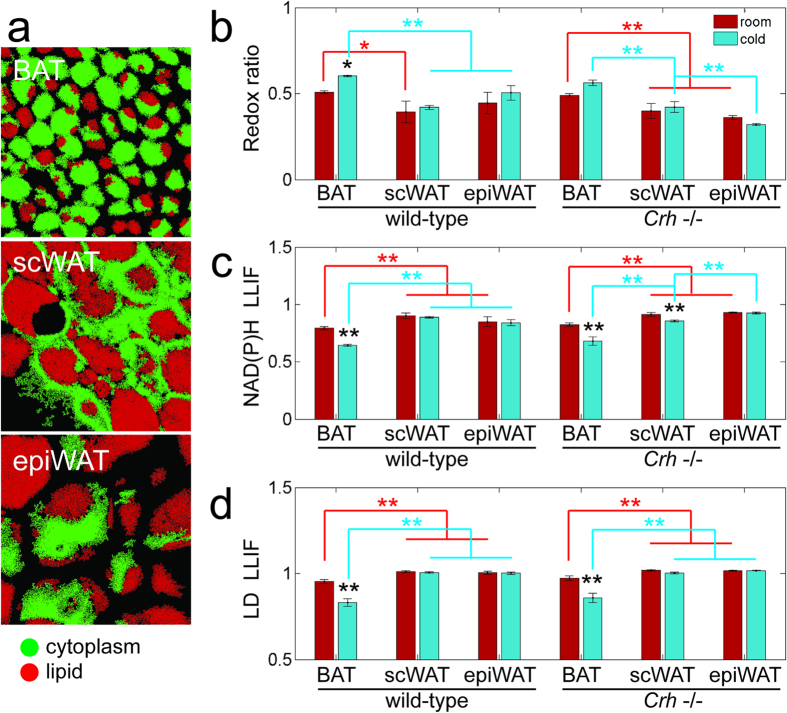
Algorithmic segmentation of images facilitates separate analysis of cytoplasm and lipid compartments. (**a**) Representative images of segmentation using combined fluorescence intensity and lifetime data (see Supplementary Fig. 3). (**b**) Mean redox ratio and (**c**) mean NAD(P)H LLIF were calculated for cytoplasmic regions, while (**d**) mean lipid LLIF was determined within lipid droplets. BAT had higher redox ratio and lower NAD(P)H and lipid fluorescence lifetimes than scWAT and epiWAT, with stronger difference after cold exposure. Cold response was also observed in scWAT of *Crh*−/− mice. Data is represented by mean ± s.e. of n = 3 mice per group. Significant differences were determined by mixed effects nested ANOVA with post-hoc Tukey HSD testing; *p < 0.05, **p < 0.001.

**Figure 6:**
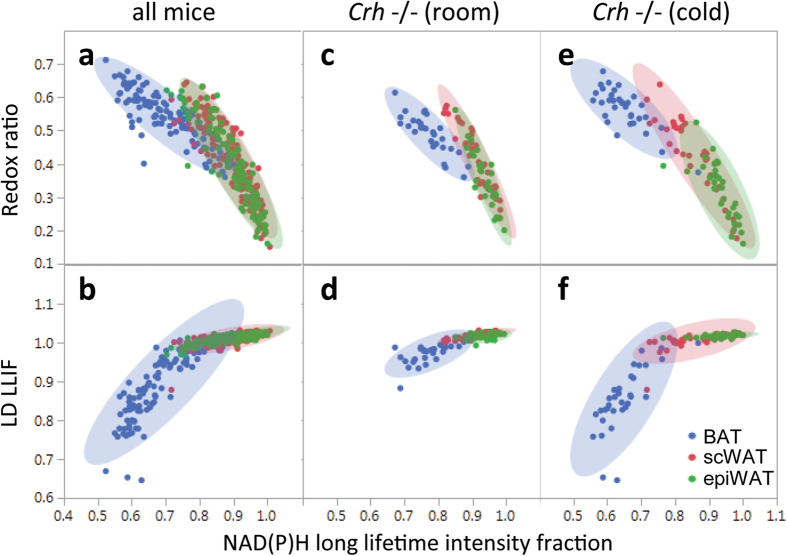
Multivariate analysis emphasizes differences between thermogenic and non-thermogenic tissue. Scatter plots depict each captured image as a separate data point, with depot groups circumscribed by 95% confidence intervals. (**a**,**b**) Pooling across all mice, BAT (blue, n = 144 images from 12 mice) follows a distinct trajectory in the multivariate space compared to the common trajectory of scWAT (red, n = 144 images from 12 mice) and epiWAT (green, n = 121 images from 12 mice) depots. We also specifically consider the cold response of *Crh*−/− mice. (**c**,**d**) At room temp, scWAT (n = 39 images from 3 mice) occupies the same parameter space as epiWAT (n = 32 images from 3 mice), separate from BAT (n = 33 images from 3 mice); (**e**,**f**) scWAT distribution (n = 36 images from 3 mice) stretches towards the BAT data points (n = 33 images from 3 mice), but still overlaps with epiWAT (n = 34 from 3 mice) – consistent with thermogenic activation of BeAT in this depot.

To further quantify differences between tissue depots, we applied algorithmic segmentation of cytoplasm and lipid compartments in each image (Fig. 5a) using combined fluorescence intensity and lifetime data (Supplementary Fig. 6, Supplementary Methods). Mean redox ratio and NAD(P)H LLIF were calculated for cytoplasmic regions (Fig. 5b,c), while a separate mean LLIF was determined for LDs (Fig. 5d). In general, BAT was characterized by significantly higher redox ratios, shorter NAD(P)H fluorescence lifetime (i.e. lower NAD(P)H LLIF) and shorter LD fluorescence lifetime, compared to scWAT and epiWAT depots. Cold exposure enhanced these differences by further increasing redox and decreasing both NAD(P)H and LD fluorescence lifetimes in BAT. For *Crh*−/− mice, the scWAT also exhibited a significant increase in redox and decrease in NAD(P)H lifetime in response to cold; consistent with scWAT browning in these mice observed via histology and RT-PCR (Supplementary Fig. 3). These trends persisted, even when the segmented cytoplasmic and LD regions were eroded by five pixels to avoid any potential signal cross-talk from the two areas (Supplementary Fig. 7). Collectively, these results demonstrate our measures are sensitive to thermogenic function, despite broad inter- and intra-tissue variability (Fig. 6). Furthermore, we observed quantifiable differences not just in the widely utilized optical redox ratio and NAD(P)H fluorescence lifetime parameters, but also in the LD intrinsic fluorescence lifetime.

### Multivariate analysis emphasizes differences between thermogenic and non-thermogenic tissue

Bivariate scatter plots of image-by-image mean redox ratio, NAD(P)H LLIF, and LD LLIF facilitate multivariate analysis and depict metabolic heterogeneity within tissues. Pooling all treatment groups, we noted strong negative correlations between redox ratio and NAD(P)H LLIF (Fig. 6a), with BAT (R = −0.87, p < 0.001) exhibiting a distinct regression slope (−0.75) compared to the combined WAT depots (slope = −1.5, R = −0.89, p < 0.001). Analysis of covariance (ANCOVA) confirmed the slopes to be significantly different (p < 0.001). Differences in linear correlation were also observed in the LD LLIF vs. NAD(P)H LLIF space (Fig. 6b). *Crh*−/− mice maintain these general trends at room temperature (Fig. 6c,d), but exhibit more distinct separation between BAT and epiWAT after cold exposure (Fig. 6e,f). Notably, *Crh*−/− scWAT exhibits a heterogeneous cold response. Certain samples exhibited increased redox ratio and decreased fluorescence lifetime more similar to thermogenic BAT, while other samples remained firmly within the non-thermogenic epiWAT parameter space. In fact, such increased variability in scWAT (Supplementary Fig. 8) was likely indicative of spatially heterogeneous browning into BeAT. These results show that combining optical redox ratio, NADH fluorescence lifetime, and LD fluorescence lifetime reveals more nuanced differences between thermogenic and non-thermogenic adipose tissue, and emphasize the complementarity of the optical measures employed. In contrast to traditional adipose tissue functional assays, imaging-based analyses account for tissue heterogeneity and can even utilize inherent variability to explore correlations that reveal important tissue differences.

### Functional TPEF imaging is translatable to *in vivo* study of adipose tissue

**Figure 7:**
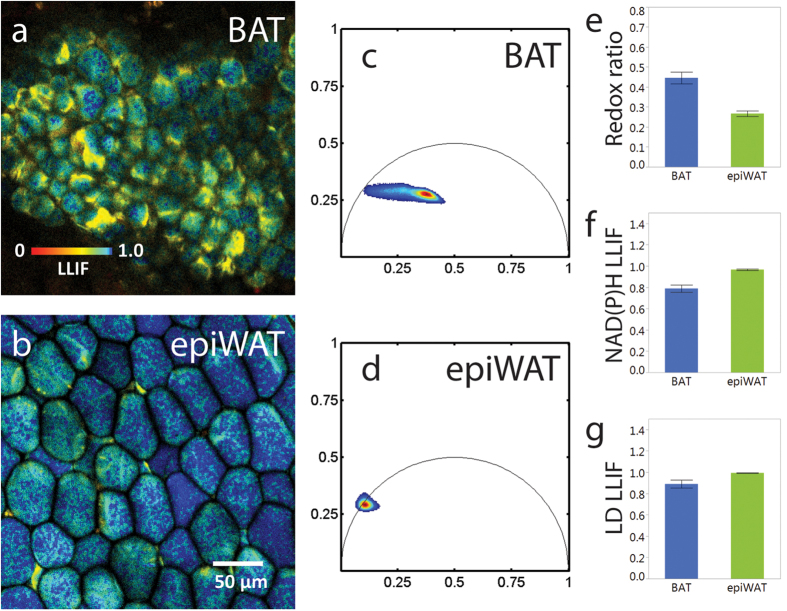
*In vivo* TPEF imaging of adipose tissue. (**a**,**b**) Fluorescence lifetime images acquired from adipose depots in live mice. (**c**,**d**) Phasor distributions (n = 5 images) from *in vivo* data show similar profiles as observed with previously frozen tissue (Fig. 4). Quantitative differences between BAT and epiWAT in (**e**) mean redox ratio, (**f**) NAD(P)H LLIF, and (**g**) LD LLIF were also consistent with trends seen in Fig. 5. Data is represented by mean ± s.e. of n = 5 images. Significant differences were determined by Student’s t-test; *p < 0.05, **p < 0.001.

We captured *in vivo* redox ratio and fluorescence lifetime images (excitation: 755 nm and emission: 460 ± 20) of BAT (n = 5 fields) and epiWAT (n = 5 fields) from separate live mice (C57BL/6, housed at room temperature) under gas anesthesia (Fig. 7a,b). We did not image scWAT as our earlier results (Fig. 5b,d) suggested that this depot would be very similar to the epiWAT in these mice. *In vivo* images featured similar contrast as those from frozen specimens (Fig. 3). Cumulative phasors from BAT occupied space within the reference arc, indicative of a bi/multiexponential decay, while the WAT phasors were more confined and had a centroid very close to the reference arc point corresponding to a 6.1 ns lifetime (Fig. 7c,d). Accordingly, both the cytoplasmic and LD regions of epiWAT had a significantly higher LLIF than BAT, while the cytoplasmic optical redox ratio was lower for epiWAT than for BAT (Fig. 7e–g). These results demonstrate that the sensitivity of the optical readouts of adipose tissue type and function we describe is translatable to *in vivo* imaging.

## Discussion

We demonstrate that different adipose tissue types have distinct optical signatures, which change upon activation of thermogenic function. Specifically, BAT from mice is characterized by a higher optical redox ratio and shorter fluorescence lifetimes for both cytoplasmic NAD(P)H and LDs compared to WAT. These differences are further enhanced by cold-induced thermogenesis in the mice. These optical biomarkers reflect the differing utilization of bioenergetic and biosynthetic pathways associated with adipose tissue function. In addition, they can be used to represent the heterogeneity of cellular function and responses, especially in the case of BeAT.

Observed trends of intrinsic coenzyme fluorescence are consistent with known metabolic pathway preferences underlying energy storage and dissipation functions in adipose tissue. For example, WAT favors de novo fatty acid synthesis. In particular, increased flux through glycolysis, pyruvate dehydrogenase complex (PDC), and tricarboxylic acid (TCA) cycle pathways relative to low ETC utilization leads to net accumulation of NADH relative to FAD, thus low redox ratio^18^. The associated long fluorescence lifetime in these tissues indicates that NADH is accumulating primarily as enzyme-bound species^20^,^22^. Meanwhile, expression of UCP1 in BAT and BeAT induces proton leak in the inner mitochondrial membrane, which is compensated by increased electron flux through the ETC^1^. This depletes enzyme-bound NADH in the mitochondria, increasing the optical redox ratio and lowering the mean fluorescence lifetime. Greater ETC flux also ramps up reactive oxygen species (ROS) generation in mitochondria. In response to elevated oxidative stress, NADPH is consumed to refresh antioxidant defenses^32^. Cold-induced thermogenesis accelerates the metabolic uncoupling of mitochondria, thus further enhancing the redox ratio value and depressing NAD(P)H fluorescence lifetime.

In addition to reporting mean differences of functional metabolic markers, an imaging approach facilitates richer multivariate analysis by finely sampling tissue heterogeneity. Figure 6 shows the continuum of metabolic profiles present in each adipose tissue depot and the overlap between tissue types. While a multivariate analysis of variance (MANOVA) may be useful for finding the optimum contrast between thermogenic and non-thermogenic tissues (Supplementary Fig. 9), examination of bivariate correlations also provides more subtle, but important, insights. In contrast to the lack of observable correlations between redox ratio and NAD(P)H fluorescence lifetime in cancer cell lines^15^, the strong correlations in our adipose tissue suggest stability between relative NAD(P)H abundance and NAD(P)H enzyme-binding profiles. Distinct regression curves between BAT and WAT likely reflect the distinct metabolic profiles underlying the correspondingly different tissue functions. Further, shifts towards higher redox ratio and shorter NAD(P)H fluorescence lifetimes caused by cold-activation of BAT and *Crh*−/− scWAT (Fig. 5) did not alter the respective bivariate relationships. In particular, the redox ratio to NAD(P)H LLIF regression slope of scWAT remained double that of BAT (−1.4 to −0.74, p < 0.001) in cold-exposed *Crh*−/− mice. The notion that BeAT does not simply adapt a BAT metabolic profile is consistent with previous reports that BeAT is different from BAT not just in location and morphology, but more fundamentally at a gene expression level^10^.

Changes in tissue heterogeneity itself may also be a useful indicator of thermogenic function. Heterogeneous recruitment of thermogenic BeAT in *Crh*−/− scWAT is signaled by increased intra tissue variability, particularly in NAD(P)H LLIF (Supplementary Fig. 8). There is also an apparent trend towards decreased variability of redox ratio and NAD(P)H LLIF in BAT in response to cold exposure. This might suggest that only a subpopulation of brown adipocytes need to be active for thermoregulation at room temperature. In contrast, full capacity for thermogenesis must be utilized at 4 °C, and the entire BAT is thermogenically active. The increase in thermogenic BAT capacity upon exposure to stimuli, such as cold but also potentially certain foods, is often referred to as “recruitment”. “Recruitment” is a concerted process that involves cell proliferation^33^,^34^, mitogenesis^35^ and increase in the mitochondrial UCP1 content, and the observed decrease in the cytoplasmic metabolic readouts of activated BAT may reflect the level of coordination of this response. The corresponding increase in lipid fluorescence lifetime, on the other hand, could be attributed to varying amounts of utilization of the energy stored in lipid droplets by the activated brown adipocytes.

Beyond the more widely utilized cytoplasmic FAD and NAD(P)H fluorescence measures, we also introduce intrinsic LD fluorescence as a novel biomarker of adipose tissue function. Significantly shorter LD fluorescence lifetime in BAT compared to WAT might be an attribute of the contrasting functions of stored lipids in each adipose tissue type. Brown adipocytes directly utilize lipids in fatty acid oxidation to drive thermogenesis^2^,^6^, while stored lipids in white adipocytes are primarily exported into circulation^3^,^36^. Comparison of lipid droplets in BAT and WAT via confocal Raman spectroscopy suggested that WAT has a greater amount of unsaturated fats compared to BAT (Supplementary Fig. 10). LC-MS/MS detailed further biochemical contrasts, such as lower overall triglycerides and greater phospholipid content in BAT, particularly after cold-exposure (Supplementary Fig. 2). These results emphasize that LDs within different adipose tissue types have distinct biochemical profiles. These biochemical differences possibly translate to biophysical differences, such as viscosity or concentration of local quenchers, which in turn drive the contrast in LD fluorescence lifetime^37^ between adipose tissue types. LD intrinsic fluorescence has been previously attributed to lipid oxidation products^26^,^27^, possibly via formation of fluorescent Schiff base molecules^38^. More precise identification of this lipid-associated fluorophore remains open and will be invaluable to informing further interpretation, such as why LD LLIF variability increases in BAT after cold exposure. We note that 3D imaging shows the fluorescence to be distributed throughout the lipid volume, limiting possible fluorophores to hydrophobic molecular species.

To emphasize the relevance of these quantitative biomarkers, we have demonstrated that functional TPEF imaging of adipose tissue is transferable to an *in vivo* setting (Fig. 7). This opens the door to longitudinal studies of dynamic adipose tissue function in animal models. Endoscopes and microprobe objectives can minimize the surgical invasiveness of TPEF microscopy in accessing fat depots in live subjects, but at the cost of lower spatial resolution^39^. However, parallel trends for fluorescence intensity ratio and fluorescence lifetime across the entire cells suggest that the optical biomarkers we have described can remain relevant even when spatial resolution is insufficient to properly segment subcellular compartments.

In summary, we have demonstrated that TPEF microscopy can quantitatively assess adipose tissue function through functional metabolic imaging with subcellular resolution using purely intrinsic contrast. Optical redox ratio, NAD(P)H fluorescence lifetime, and LD fluorescence lifetime can distinguish energy-storage driven WAT from energy-dissipative BAT, and are further sensitive to thermogenic activation in the latter. These optical biomarkers provide the basis of a much needed toolset for non-invasive dynamic assessment of adipose tissue function towards developing interventions for metabolic health in the context of obesity, and towards elucidating deeper insights into the multifaceted physiological functions of the adipose organ.

## Materials and Methods

### Mice

Eight-week-old *Crh*−/− and wild-type male mice of C57BL/6 genetic background were housed in individual cages and acclimated at 18 °C for 2 days followed by cold exposure at 4 °C for another 2 days with a 12-hour light-dark cycle and free access to standard laboratory pellet formula and tap water, based on previously established protocols for BeAT formation and induction of UCP1 protein/gene expression levels^29^ that are well-tolerated by the animals. Control groups for both genotypes were housed at room temperature (22–25 °C) over the same period. C57BL/6 WT mice were used as negative controls for BeAT formation to compare with Crh−/− mice, which act as a positive control, as they have markedly increased susceptibility to beiging. In addition, C57BL/6J is the most widely used inbred strain and used in a wide variety of fields including diabetes and obesity, cardiovascular and developmental biology.

A dozen mice, three per genotype and treatment, were euthanized via isoflurane. Adipose tissue was excised from the interscapular, subcutaneous inguinal, and epididymal fat depots of each mouse, snap frozen with dry ice, and stored at −80 °C until imaging. All animal housing, care, and experimental procedures were approved by the Animal Care and Use Committee of the Biomedical Research Foundation of the Academy of Athens in Athens, Greece, in accordance with NIH guidelines.

### Two-photon excited fluorescence instrumentation

TPEF images were collected on a custom-built multiphoton microscope (Supplementary Fig. 11). Laser emission from a mode-locked Ti:sapphire laser (Spectra Physics, MaiTai) was focused by a 40 × 1.1 NA infrared-optimized water-immersion objective lens (Leica, HC PL IRAPO) to excite NAD(P)H and LD fluorescence at 755 nm and FAD fluorescence at 860 nm with average laser powers of 40 mW and 27 mW, respectively. Fluorescence emission was collected through the same objective and spectrally separated by a sequence of dichroic mirrors and filters (Chroma; 700DCXRU, ET650SP-3P, 500DCXRU). Optical signals were detected by a pair of GaAsP photomultiplier tubes (PMT; Hamamatsu, H7422P-40) with fixed voltage gains, each behind a bandpass filter for isolating either NAD(P)H/LD(Chroma, HQ460/40m-2P) or FAD (Chroma, HQ525/50m-2P) fluorescence, and coupled to a time correlated single photon counting system (Becker & Hickl, SPC-150). Photon count rates were in the range of 2 × 10^4^ to 1 × 10^6^, against a maximum background rate of 2 × 10^3^. Each 512 × 512 pixel image (0.36 μm/pixel) was collected over a 120 s integration time with 0.1 μs pixel dwell time.

### Imaging procedure

Adipose tissue samples were thawed at room temperature and hydrated in phosphate-buffered saline. A coverglass was placed over the specimen to maintain hydration over the imaging period. Fluorescence images at each excitation/emission channel were recorded sequentially at 6 to 12 locations in each sample, to a maximum depth of 100 μm beneath the tissue surface. Regions that exhibited strong second-harmonic generation were excluded from image acquisition to minimize the influence of extracellular matrix. Total imaging time per sample did not exceed 90 minutes. Time-resolved fluorescence from a standard 7-hydroxycoumarin solution was measured prior to each imaging session to monitor day-to-day consistency of instrumentation. Instrument impulse response was measured using second harmonic generation in urea crystals.

### *In vivo* imaging

BAT and epiWAT depots were surgically exposed in 10 week-old C57BL/6 mice under isoflurane anesthesia. Two different mice were used, i.e. one for each adipose depot, and five fields were imaged from each depot using a 25 × 0.95 NA infrared-optimized water-immersion objective lens (Leica,HCX IRAPO L) with 2.5 mm working distance. Mouse body temperature was maintained with a custom heated stage. Motion artifacts were minimized by gluing peripheral tissue to a coverglass with cyanoacrylate-latex. Care was taken to exclude glue from the imaged regions of interest. Mice were sacrificed 90 minutes after initial anesthesia induction. Animal care and experiment protocols relevant to *in vivo* imaging in mice were approved by the Institutional Animal Care and Use Committee of Tufts University, in accordance with NIH guidelines.

### Digital image analyses of optical redox ratio and fluorescence lifetime

Total photon counts at each pixel were normalized by the square of incident laser power to represent fluorescence intensity. Optical redox ratio was calculated by taking the ratio of mean fluorescence intensities within the segmented cytoplasm regions as: redox ratio = FAD/(NAD(P)H+FAD). This form has the advantage of being inherently bound between 0 and 1 and was established as a robust indicator of redox state in seminal studies performed by Chance^40^. More recently, we have demonstrated that this optical redox ratio is strongly correlated to LC-MS/MS based assessments of both FAD/(NADH+FAD) and NAD^+^/(NADH+NAD^+^) concentration ratios^18^,^19^. NAD(P)H and LD fluorescence decay profiles were subject to a phasor transform as described by Stringari *et al.*^23^. Fourier sine and cosine transforms mapped the time-resolved signal accumulated from 5-by-5 pixel bins about each image pixel to 2D phasor space (Supplementary Methods). 7-hydroxycoumarin fluorescence was used as a reference to correct for the instrumentation impulse response^41^. Phasors from all images were accumulated by group to construct normalized density maps. For image segmentation, low-intensity regions (such as nuclei and other weakly fluorescent cellular compartments) were excluded via Otsu thresholding, while a combination of FAD intensity and NADH fluorescence lifetime was used to identify cytoplasm and lipid compartments in cells (see Supplementary Methods).

### Statistical Analysis

Results from each genotype were analyzed separately, with differences in redox ratio and fluorescence lifetime assessed by mixed effects nested ANOVA of per image averages using JMP 12 (SAS Institute). The model design considered adipose tissue depot and temperature treatments as fixed effects, with individual mice as random effects nested within each temperature treatment; temperature and depot effect interactions were included. Post-hoc Tukey HSD tests were used to evaluate differences within each effect. Pearson’s correlation coefficients, regression slopes, ANCOVA for evaluating differences between slopes, and MANOVA were calculated using MATLAB (Mathworks).

## Additional Information

**How to cite this article**: Alonzo, C. A. *et al.* Two-photon excited fluorescence of intrinsic fluorophores enables label-free assessment of adipose tissue function. *Sci. Rep.* **6**, 31012; doi: 10.1038/srep31012 (2016).

## Supplementary Material

Supplementary Information
