# Supplementary material for: Two-photon excited fluorescence of intrinsic fluorophores enables label-free assessment of adipose tissue function
Source: Sci Rep. 2016 Aug 5;6:31012. doi: 10.1038/srep31012 (PMC4974509; doi:10.1038/srep31012)
Supplement: Supplementary Information [file srep31012-s1.pdf]

Classification: BIOLOGICAL SCIENCES – Applied Biological Sciences

Title: Two-photon excited fluorescence of intrinsic fluorophores enables label-free assessment of adipose tissue function

Authors:
Carlo Amadeo Alonzo[1], Sevasti Karaliota[2], Dimitra Pouli[1], Zhiyi Liu[1], Katia P. Karalis[2,3], Irene Georgakoudi[1]
[1]Department of Biomedical Engineering, Tufts University, Medford, MA 02155
[2]Biomedical Research Foundation, Academy of Athens, Athens, Greece
[3]Endocrine Division, Children's Hospital, Boston, MA 02115

Corresponding Author:
Irene Georgakoudi
4 Colby St.
Dept. of Biomedical Engineering
Tufts University, Medford, MA 02155
(617) 627-4353
Irene.Georgakoudi@tufts.edu

Keywords: imaging, metabolism, adipose tissue, brown adipose tissue

Supplementary Information

Supplementary Figures

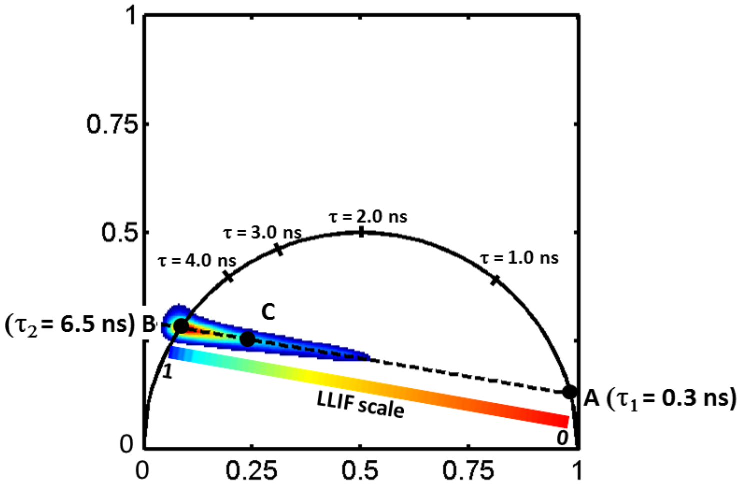

Supplementary Fig. 1. Phasor analysis of fluorescence lifetime. Fourier sine and cosine transforms map the decay curve at each pixel of an image to 2D phasor space (see supplementary methods below). Monoexponential decays, exp(-t/τ), fall onto a reference semi-circle, with τ -> 0 at (1,1) and τ -> ∞ at (0,0). Tick marks illustrate that τ does not distribute linearly along this arc. Biexponential decays, A_1exp(-t/τ_1)+A_2exp(-t/τ_2), will fall along a line that passes through the corresponding components on the reference arc. Fluorescence decays measured from our adipose tissue samples followed a trajectory from τ_1 = 0.3 ns (A) to τ_2 = 6.5 ns (B). For a phasor located at point C, the ratio of the segment AC over the total length of AB represents the fractional intensity contribution of the long lifetime component, τ_2,to the total fluorescence at that point: $A_2\tau_2/(A_1\tau_1+A_2\tau_2)$. Conversely, BC/AB = $A_1\tau_1/(A_1\tau_1+A_2\tau_2)$. For fluorescence lifetime images (Figs. 1 and 3), we calculate the long lifetime intensity fraction (LLIF) at each point and assign a color scale as depicted above. For phasors that do not fall exactly on the assigned trajectory, LLIF is based on a perpendicular projection to the line segment.

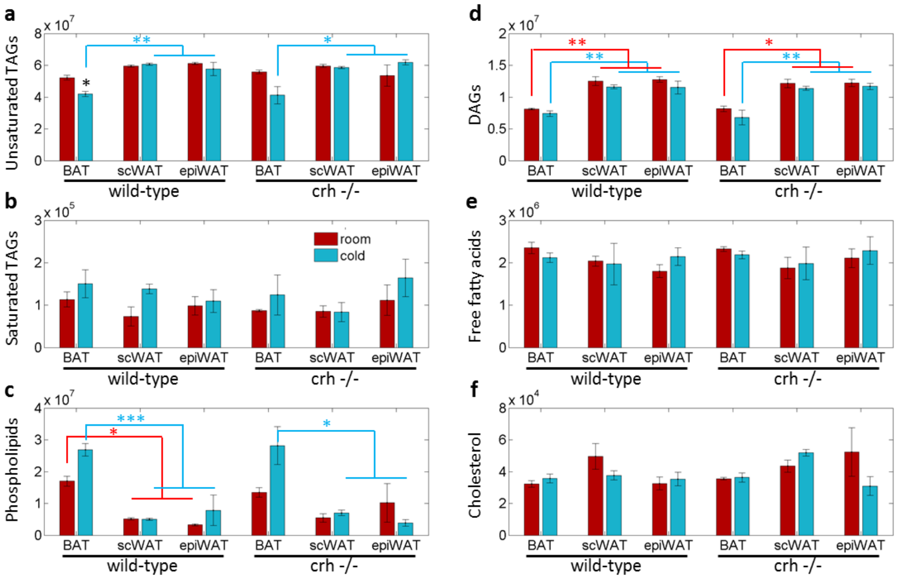

Supplementary Fig. 2. Summary of lipidomic analysis via LC-MS/MS. Individual molecular lipid species were quantified by mass chromatogram peak height, normalized to the total ion chromatogram. Identified species were then grouped by lipid class, namely (a) unsaturated triacylglycerols (TAGs), (b) saturated TAGs, (c) phospholipids, (d) diacylglycerols (DAGs), (e) free fatty acids, and (f) cholesterol. Cold-activation of thermogenesis in BAT raises phospholipid levels due to mitochondrial biogenesis, while TAG levels fall due to increased rates of beta oxidation. Data is represented by mean ± s.e. of n = 3 mice per group. Significant differences were determined by mixed effects nested ANOVA with post-hoc Tukey HSD testing; *p<0.05, **p<0.005, ***p<0.001.

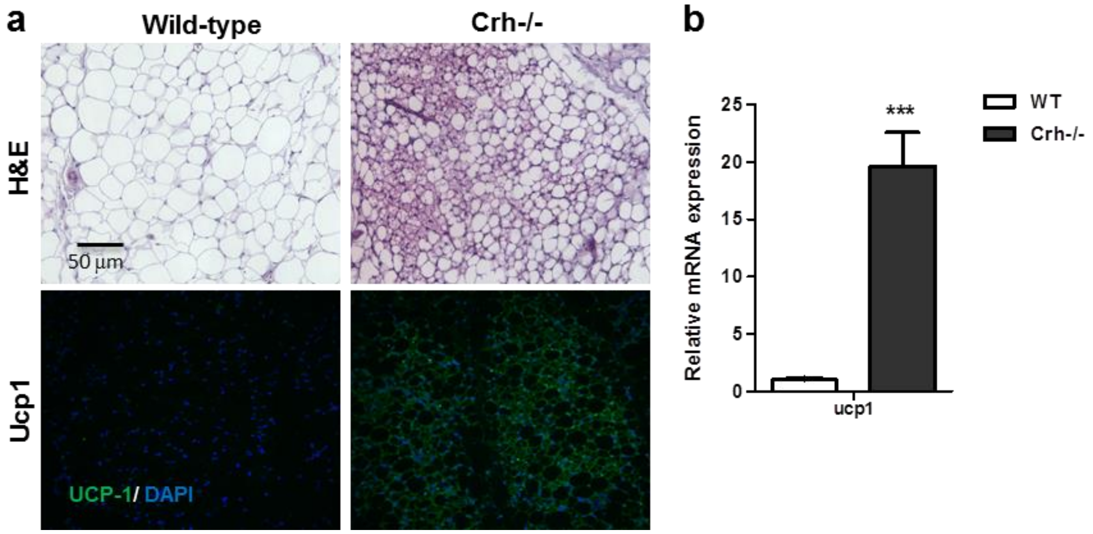

Supplementary Fig. 3. Thermogenic BeAT is induced in scWAT of *Crh*-/- mice, but not in wild-type mice. (a) H&E histological sections show smaller lipid droplets are present in scWAT of *Crh*-/- mice compared to wild-type; UCP1 immunofluorescence staining shows rich prevalence of the thermogenesis enabling uncoupling protein in scWAT of *Crh*-/- mice. (b) *UCP1* gene expression shows ten-fold increase in Crh-/- scWAT compared to wild-type. Data is presented as mean ± s.e. of n = 5 mice per group. Significance value is at ***p<0.001 by Student's t-test. Description of histological analysis and quantitative RT-PCR are provided in Supplementary Methods.

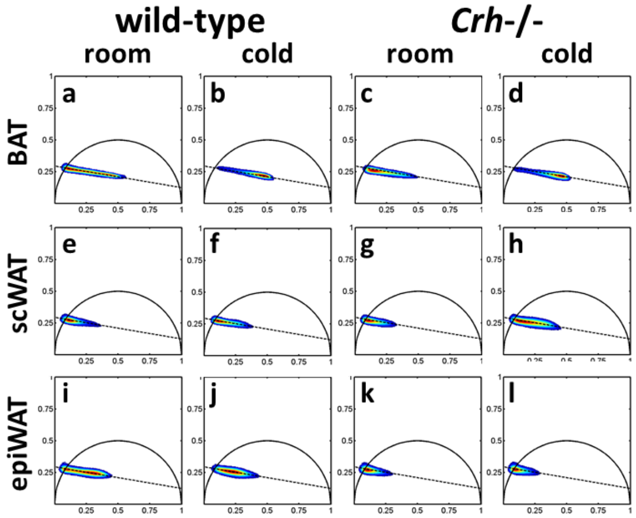

Supplementary Fig. 4. Phasor distribution from segmented cytoplasm compartments. After digital segmentation (Supplementary Methods), we calculated phasor distributions maps corresponding to cytoplasmic regions. Exclusion of LD fluorescence contributions reveals that cytoplasm in scWAT and epiWAT also feature broad distributions along the same linear trajectory as BAT. Each panel depicts peak normalized phasor distribution averaged from 3 mice, based on 6 to 15 acquired images per mouse.

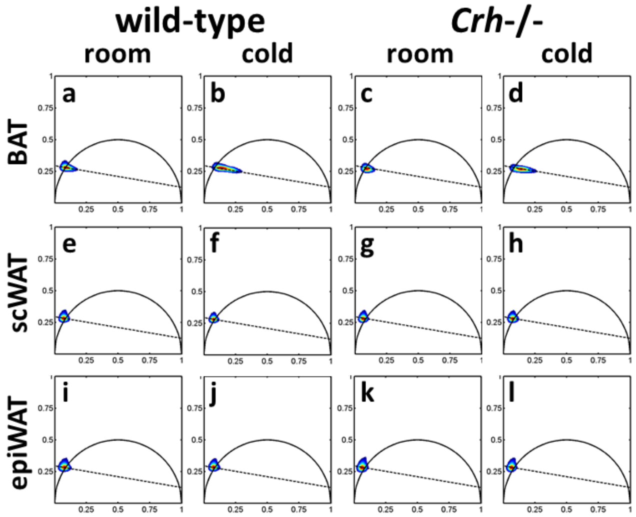

Supplementary Fig. 5. Phasor distributions from segmented lipid compartments. Isolation of LD fluorescence via digital segmentation (Supplementary Methods) shows tight clustering of phasors about τ = 6.5 ns on the reference arc for scWAT and epiWAT. This strongly suggests that LD fluorescence is characterized by monoexponential decay in these depots. In contrast, BAT LDs present distributions that stretch below the arc, indicative of multiexponential profiles. Each panel depicts peak normalized phasor distribution averaged from 3 mice, based on 6 to 15 acquired images per mouse.

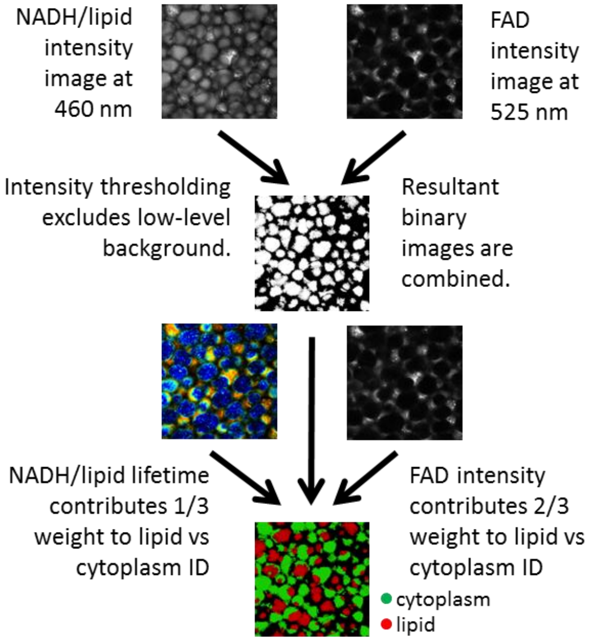

Supplementary Fig. 6. Algorithmic image segmentation of cytoplasm and lipids takes into consideration both spectral fluorescence intensity and lifetime information. Digital image segmentation starts with excluding low-intensity regions. Nuclei are weakly fluourescent and tend to be excluded by this step as well.. Based on cell morphology, we noted that lipids exhibited low fluorescence intensity in the FAD channel and long fluorescence lifetime in the NADH channel. We weighted these two criteria in assigning each pixel in an image either a cytoplasm or lipid label. See Supplementary Methods for more detail.

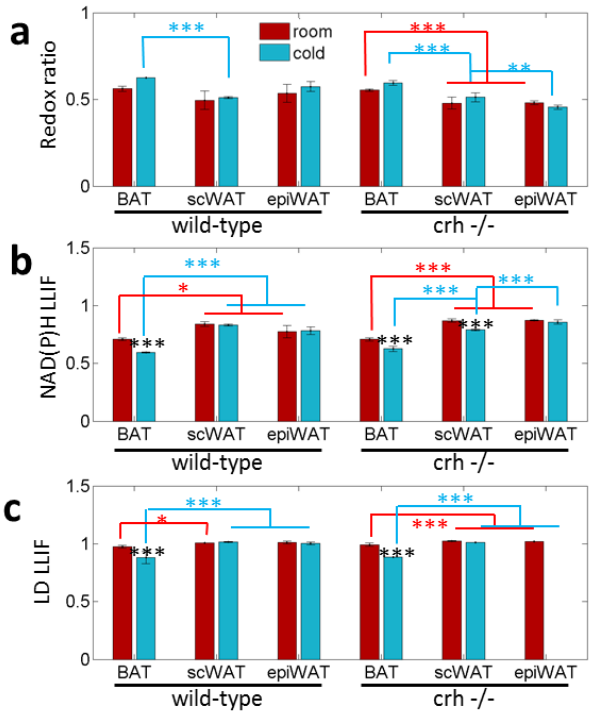

Supplementary Fig. 7. Mean redox ratio, NAD(P)H LLIF, and LD LLIF after erosion of segmentation masks. Digital segmentation masks were eroded by 5 pixels along the perimeters of both cytoplasmic and lipid regions to minimize the influence of possible crosstalk between regions. Trends observed in Fig. 5 persisted even under these stricter regions of interest. In general, BAT had higher redox ratio and lower NAD(P)H and lipid fluorescence lifetimes than scWAT and epiWAT, with stronger difference after cold exposure. Cold response was also observed in scWAT of Crh-/- mice. Data is represented by mean ± s.e. of n = 3 mice per group. Significant differences were determined by mixed effects nested ANOVA with post-hoc Tukey HSD testing; *$p<0.05$, **$p<0.005$, ***$p<0.001$.

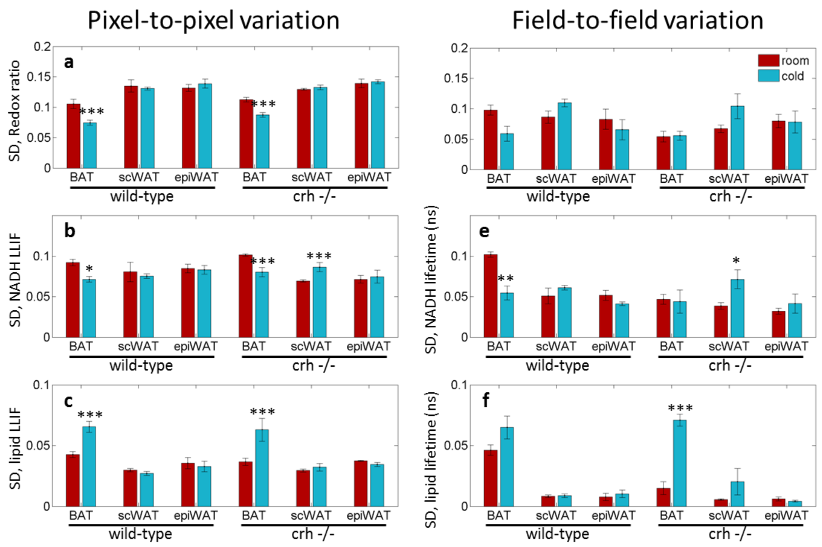

Supplementary Fig. 8. Average standard deviation (SD) measures tissue heterogeneity at different length scales. (a-c) Micrometer-scale heterogeneity is measured as SD of pixels within each image averaged across images, then across mice. Significant decrease in pixelwise variability of redox ratio and NADH fluorescence lifetime, and increase in lipid fluorescence lifetime variability appears to be a mark of cold-activated thermogenesis in BAT. Notably, scWAT in *Crh-/-* mice shows significantly increased variability in NADH fluorescence lifetime with cold exposure. (d-f) Millimeter-scale heterogeneity is evaluated by SD of image mean values within each mouse averaged across mice. Similar trends in variability were observed as at the finer spatial scale. . Data is represented by mean ± s.e. of n = 3 mice per group, 6 to 15 images per mouse, and 512 x 512 pixels per image. Significant differences were determined by mixed effects nested ANOVA with post-hoc Tukey HSD testing; *p<0.05, **p<0.005, ***p<0.001.

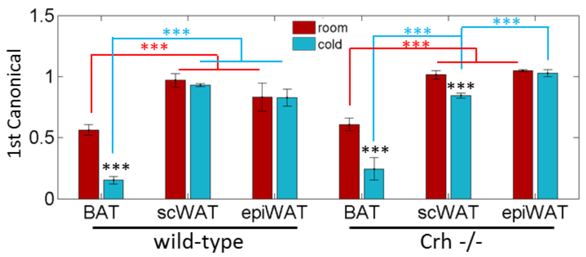

Supplementary Fig. 9. Multivariate analysis maximizes contrast between groups. We used multivariate analysis of variance (MANOVA) to calculate the 1st canonical vector that maximized contrast between BAT and WAT tissue after cold activation. The derived canonical was C1 = 0.4 redox ratio + 3.0 NADH LLIF – 0.3 lipid LLIF – 2.2. Data is represented by mean ± s.e. of n = 3 mice per group. Significant differences were determined by mixed effects nested ANOVA with post-hoc Tukey HSD testing; ***p<0.001.

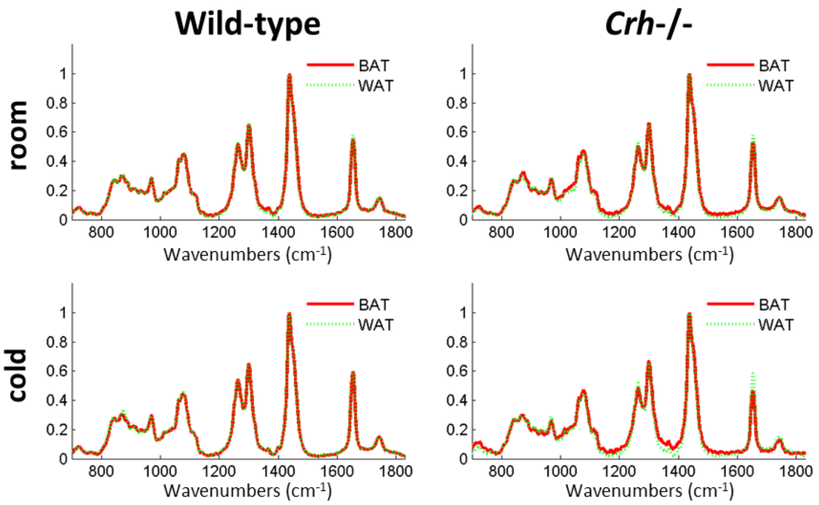

Supplementary Fig. 10. Confocal Raman spectra of lipid droplets in BAT and WAT are very similar. We isolated Raman spectra originating from lipid droplets in adipose tissue (see supplementary methods). Typical lipid features are well represented, including acyl CH_2,CH_3 bend at 1440 cm^{-1} and C=C stretch at 1660 cm^{-1}, ester C=O stretch at 1750 cm^{-1}; spectra are normalized to the 1440 cm^{-1} peak. The most observable contrast between BAT and WAT is at 1660 cm^{-1}, suggesting greater fatty acid saturation levels in BAT. Further characterization, e.g. HPLC and lipidomic profiling, may provide more useful clues to the origin of the intrinsic lipid fluorescence and the observed differences in lipid fluorescence lifetime between BAT and WAT.

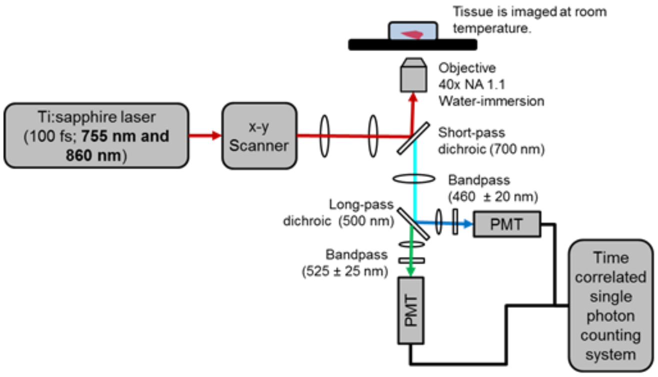

Supplementary Fig. 11. TPEF microscopy is performed on a custom-built microscope equipped for multiphoton excitation and time-correlated single photon counting detection (TCSPC). Transverse digital sampling resolution is 0.36 μm/pixel with a 40x objective lens (Leica, HC PL IRAPO). Fluorescence signals were detected with a pair of GaAsP photomultiplier tubes (PMT; Hamamatsu, H7422P-40) coupled to a TCSPC system (Becker & Hickl, SPC-150) enabling time-resolved measurement. Photon count rates were in the range of 2 x 10^4 to 1 x 10^6, against a maximum background rate of 2 x 10^3. Each 512 x 512 pixel image (0.36 μm/pixel) was collected over a 120 s integration time with 0.1 μs pixel dwell time.

Supplementary Methods

Histological analysis. Tissues were dissected and fixed in 4% paraformaldehyde and processed for routine paraffin histology. Paraffin-embedded tissues were sectioned at 5µm and stained with Hematoxylin and Eosin (H&E) according to standard protocol. Images were obtained using a brightfield LEICA DMLS2 microscope. For immunofluroscence staining of Ucp-1, a rabbit anti-Ucp1 antibody were used (1:300, ab10983, Abcam). Images were obtained using a confocal inverted LEICA TCS SP5 (DMI6000).

Quantitative Real-Time RT-PCR. Total RNA was isolated from tissues using TRI reagent (Sigma) and treated with DNase using the DNA-free kit (Ambion). Complemetary DNA was made from 2µg total RNA by MMLV reverse transcriptase (Invitrogen) and initiated from random hexamer primers (Life Technologies Inc). Quantitative real – time PCR analysis was performed using RT² SYBR® Green qPCR Master Mix (SA biosciences) in ABI PRISM 7000 Sequence Detection System (Applied Biosystems). Primers used for real-time PCR, UCP1: 5'-TCTTCTCAGCCGGAGTTTCAGCTT-3' and 5'-ACCTTGGATCTGAAGGCGGACTTT-3. Gene expression levels were normalized to actin and calculated according to the 2-ΔΔCt method.

Phasor analysis of fluorescence lifetime. Following Stringari et al. [1], we applied the phasor transform to time-resolved fluorescence data. In general, phasor analysis offers intuitive visualization of subtle differences in complex decay profiles. Fourier sine and cosine transforms map each image pixel in acquired images to two-dimensional phasor space. Phasors from pure monoexponential decays fall on a unit radius semi-circle passing (0,0) and (1,1), often referred to as the "universal circle". Decay constants, i.e. fluorescence lifetime τ, increases in the counter-clockwise direction along this reference arc; with τ -> 0 at (1,1) and τ -> ∞ at (0,0). Multiexponential decays fall below the universal circle. In particular, a linear trajectory in phasor space suggest biexponential decays with decay components corresponding to the intersections of the linear trajectory and the universal circle. Further, the fractional contribution of each component to total fluorescence intensity is proportional to the length of each segment connecting the phasor to the opposite reference point. Notably, the instrument impulse response of a fluorescence lifetime imaging system introduces rotation and radial modulation to a phasor with respect to the origin [2]. We used 7-hydroxycoumarin fluorescence (excitation: 755 nm, emission: 460±20nm, τ = 5.1 ns) as a reference to correct for the instrument impulse response. To improve the signal to noise, 5-by-5 pixel binning was applied as phasors were calculated. Phasors from each image were then accumulated by group to construct normalized density maps representing each combination of genotype, tissue depot, and temperature treatment. To calculate the long lifetime intensity fraction (LLIF) at each pixel, we took the perpendicular projection of each phasor to a line intersecting the universal circle at τ = 0.3 ns and τ = 6.5 ns and measured the distance to τ = 0.3 ns, then divided by the full length of the reference line.

Image segmentation algorithm. Three-level Otsu intensity thresholding [3] was applied to each fluorescence image with the lowest level designated as low intensity background noise or weakly fluorescent cell compartments (e.g. nuclei). Regions assigned to the upper two quantized levels in corresponding NADH and FAD images were combined to define the complete cell or tissue area. These

cell regions were further segmented into cytoplasm and lipid droplet compartments by combining fluorescence intensity and lifetime information. Specifically, a lipid probability score was calculated for each pixel according to

$$P_{lipid} = \frac{2}{3}\left[1 - \min\left(\frac{I_{FAD}}{threshold_{FAD}}, 1\right)\right] + \frac{1}{3}\left[\min\left(\frac{\tau_m}{\tau_{cutoff}}, 1\right)\right]$$

where I_{FAD} is FAD fluorescence intensity, $threshold_{FAD}$ is the lower Otsu threshold, τ_m is mean fluorescence lifetime in the NADH channel, and τ_{cutoff} is a reference lifetime set to 6 ns. If $P_{lipid} > 0.5$, then the pixel is classified as lipid, otherwise it is labeled as cytoplasm.

Raman spectra of lipid droplets. Spontaneous Raman scattering spectra from adipose tissue samples were measured using a custom-built confocal Raman microscopy system at the MIT Laser Biomedical Research Center [4]. Briefly, a continuous wave Ti:sapphire laser (Spectra-Physics, 3900s,) delivered 785 nm excitation through a 60x 1.2 NA infrared-optimized water-immersion objective lens (Olympus, UPLSAPO60XWIR). Backscatter emission was collected by the same objective, filtered through a pair identical dichroic mirrors (Semrock, LPD01-785RU), then coupled to an imaging spectrograph (Kaiser Optical Systems, HoloSpec f/1.8i) via a multimode optical fiber (Thorlabs, M14L01) and detected by a TE-cooled, back-illuminated, deep depleted CCD (Princeton Instruments, PIXIS 100BR eXcelon). Raman spectra up to 1830 cm^{-1} were recorded for 0.1 s at each pixel in a 30 x 30 array over a 150 μm x 150 μm field of view at 3 location in each sample. High signal intensity at 1440 cm^{-1} (CH_2 and CH_3 bend) was used to identify lipid droplets and segment via Otsu thresholding. Average spectra across lipid pixels were normalized to this peak; background was estimated by fitting to a fifth-order polynomial, then subtracted.

LC-MS/MS Lipidomic analysis

Frozen tissue samples were held at -80°C and sent to the NIH West Coast Metabolomics Center at the University of California, Davis. Lipid fractions were extracted from tissue homogenates using acetonitrile, isopropanol, and water (3:3:2). For each run, 3 μL of reconsituted extracts were injected in a C18 column (Waters, Acquity UPLC CSH C18). Gradient elution of water:acetonitrile (40:60) to isopropanol:acetonitrile (90:10) was applied at a flow rate of 0.6 ml/min . Excellent retention and separation of lipid classes was demonstrated with narrow peak widths of 8-17 s. Within-series retention time reproducibility was better than 6 s absolute deviation. Positively charged lipids were analyzed with an Agilent 6530 QTOF mass spectrometer with resolution R = 10000, while negatively charged lipids were resolved with an Agilent 6550 QTOF mass spectrometer (R = 20000). Raw data was first processed in an untargeted manner by MassHunter Qual software (Agilent) to find peaks. Peak features were then aligned using MassProfilerProfessional. Peaks appearing in less than 30% of samples were excluded. MS/MS information of identified peaks is then compared to the LipidBlast library to identify specific lipids. Quantitative comparisons are based on peak heights, normalized to the total ion chromatogram of identified metabolites.

Supplementary References

1 Stringari, C. *et al.* Phasor approach to fluorescence lifetime microscopy distinguishes different metabolic states of germ cells in a live tissue. *Proceedings of the National Academy of Sciences of the United States of America* **108**, 13582-13587, doi:10.1073/pnas.1108161108 (2011).

2 Martelo, L., Fedorov, A. & Berberan-Santos, M. N. Fluorescence Phasor Plots Using Time Domain Data: Effect of the Instrument Response Function. *J Phys Chem B* **119**, 10267-10274, doi:10.1021/acs.jpcb.5b00261 (2015).

3 Liao, P. S., Chew, T. S. & Chung, P. C. A fast algorithm for multilevel thresholding. *J Inf Sci Eng* **17**, 713-727 (2001).

4 Kang, J. W. *et al.* Combined confocal Raman and quantitative phase microscopy system for biomedical diagnosis. *Biomedical optics express* **2**, 2484-2492, doi:10.1364/BOE.2.002484 (2011).
